# Supplementary material for: Mode of Action of Brassinosteroids: Seed Germination and Seedling Growth and Development—One Hypothesis
Source: Int J Mol Sci. 2025 Mar 12;26(6):2559. doi: 10.3390/ijms26062559 (PMC11942388; doi:10.3390/ijms26062559)

SUPPLEMENT DATA

1. **Introduction** (not ilustrations)

2. **Observation regarding the applied research methodology** (not ilustrations)

3. **Germination of a seeds of different plant species, early phases of a seedling growth and development and some processes affecting seedling growth and development under the influence of different brassinosteroids**

Table S1. Effect of different concentrations of various BRs with or without additional treatments on germination parameter (%) of the plant species/genotypes/ecotypes.

Type of BRs	Concentration of BRs	Other Treatments	Plant species or/ and genotypes/ ecotype; age of seedlings/ plants	Measured parameters (with unit of measure)	higher (>, ≥)/ lower (<, ≤) values then control or with non-significant difference (n-s.d.)	References
24-EBL	from 5.2 X 10^{-7} to 5.2 X 10^{-15} M	-	*maize* ZP434/ZP704) 7 d old	Germination (%)	< 5.2 X 10^{-7} M	[22]
24-EBL	-II-	-	*maize* ZP434/ZP704) 7 d old	Germination (%)	n-s.d. 5.2 X 10^{-15} M	[22]
24-EBL	-II-	-	*maize* (ZP434) 7 d old	Germination (%)	> 5.2 X 10^{-12} M	[21]
24-EBL	-II-	-	*maize* (ZP704) 7 d old	Germination (%)	n-s.d. 5.2 X 10^{-12} M	[21]
24-EBL	-II-	-	*maize* (ZP434) 7 d old	Germination (%)	< 5.2 X 10^{-9} M	[21]
24-EBL	-II-	-	*maize* (ZP704) 7 d old	Germination (%)	< 5.2 X 10^{-9} M	[21]
24-EBL	10^{-8} M	BZR, 50 mM snd 100 mM of NaCl	*A. thaliana* (ecotype Columbia 0) 21 d old	Germination (%)	n-s.d. 10^{-8} M	[23]
24-EBL	2.5 X 10^{-6} M	GA mutant *ga3-1, ga2-1* of *A. Thaliana*	*A. thaliana* (ecotypes Landsburg erecta and Columbia) 5 d old	Germination (%)	n-s.d. 2.5 X 10^{-6} M	[24]
24-EBL	2.5 X 10^{-6} M	GA mutants *ga1-3, sly1-2* of *A. Thaliana*	*A. thaliana* (ecotypes Landsburg erecta and Columbia) 5 d old	Germination (%)	< 2.5 X 10^{-6} M	[24]
28-HBL/ 24-EBL	10^{-6}, 10^{-8} and 10^{-10} M	Natural conditions	*Brasicca juncea* 10 d old	Germination (%)	≥ 10^{-6} M	[25]
24-EBL	10^{-8} M	H_2O_2+cold (4^0C, 3 h during 3 d)	*Brasicca juncea* 10 d old	Germination (%)	n-s.d. 10^{-8} M	[26]
28-HBL/ 24-EBL	0.5, 1.0, 2.0 10^{-6} M	PEG6000 (15% dilution) induced drought	*Raphanus sativus* 3+7 d old	Germination (%)	≤ 1.0 10^{-6} M	[27]

Table S2. Effect of different concentrations of various BRs with or without additional treatments on different seedling growth parameters of a various plant species/genotypes/ecotypes.

Type of BRs	Concentration of BRs	Other Treatments	Plant species or/ and genotypes/ ecotype; age of seedlings/ plants	Measured parameters (with unit of measure)	higher (>, ≥)/ lower (<, ≤) then control or with non-significant difference (n-s.d.)	References
24-EBL	from 5.2 X 10^{-7} to 5.2 X 10^{-15} M	-	*maize (*ZP434,ZP704) 7 d old	Shoot (plumula) length (cm)	< 5.2 X 10^{-7} M	[22]
24-EBL	-II-	-	*maize* (ZP434) 7 d old	Shoot length (cm)	> 5.2 X 10^{-15} M	[22]
24-EBL	-II-	-	*maize* (ZP704) 7 d old	Shoot length (cm)	n-s.d. 5.2X10^{-15} M	[22]
24-EBL	-II-	-	*maize* (ZP434) 7 d old	Shoot length (cm)	> 5.2 X 10^{-15} M	[21]
24-EBL	-II-	-	*maize* (ZP704) 7 d old	Shoot (plumula) length (cm)	n-s.d. 5.2X10^{-15}M	[21]
24-EBL	-II-	-	*maize* (ZP434/ZP704) 7 d old	Shoot length (cm) Relative mass (g/g) of shoot (SMR) and root (RMR)	< 5.2 X 10^{-7}, 5.2 X 10^{-9} M	[21]
24-EBL	-II-	-	*maize* (ZP434/ZP704) 7 d old seedlings	Root length (cm) Relative mass (g/g) of shoot (SMR) and root (RMR)	n-s.d. 5.2 X 10^{-9}, 5.2 X 10^{-15} M	[21]
24-EBL	10^{-6}, 10^{-8} and 10^{-10} M	Heat (40^0C, 3 h during 3 d)	*Brassica juncea* L. 10 d old	Shoot length (cm)	< 10^{-6} M	[28]
24-EBL	10^{-6}, 10^{-8} and 10^{-10} M	Heat (40^0C, 3 h during 3 d)	*Brassica juncea* L. 10 d old	Root length (cm)	n-s.d. 10^{-6} M	[28]
24-EBL	10^{-5}, 10^{-6} and 10^{-7} M	Cold (~20/5^0C, day/night)	*maize* (line LM-17) 7, 14, 21 d after 1st leaf stage seedlings	Shoot length (cm) and fresh mass (g)	< 10^{-6} M	[29]
24-EBL	10^{-5}, 10^{-6} and 10^{-7} M	Cold (~20/5^0C, day/night)	*maize* (line LM-17) 7, 14, 21 d after 1st leaf stage seedlings	dry plant mass (g)	n-s.d. 10^{-6} M	[29]
24-EBL	10^{-7} M	Chlorpyrifos (0,06% dilution)	*rice* (indica type) 12 d old	fresh shoot/ root and total plant mass (g)	≥ 10^{-7} M	[30]
28-HBL	10^{-9} M	Heat (40^0C)/ cold (4^0C)/ salt (180 mM)	*Brasicca juncea* 10 d old	Shoot/root length (cm) and fresh/ dry mass (mg)	n-s.d. 10^{-9} M	[31]
28-HBL/ 24-EBL	10^{-6}, 10^{-8} and 10^{-10} M	Natural conditions	*Brasicca juncea* 10 d old	Shoot length (cm)	≥ 10^{-6} M	[25]

24-EBL	10^{-8} M	H_2O_2, cold	*Brasicca juncea* 10 d old	Shoot/root length (cm)	< 10^{-8} M	[26]
28-HBL/ 24-EBL	0.5, 1.0, 2.0 10^{-6} M	PEG6000 (15%) induced drought	*Raphanus sativus* 3+7 d old	Shoot legth (cm) and fresh/ dry mass (mg)	< 1.0 10^{-6} M	[27]
28-HBL	10^{-7}, 10^{-9} and 10^{-11} M	Zn (different concentrations)	*Brasicca juncea* 7 d old	Shoot/root legth (cm)	< 10^{-7} M	[32]
24-EBL	10^{-6} - 10^{-9} M	DHECD (BRs analog; various concentrations) +heat (47^0C)	*rice* (indica type) 35+7+7 d	fresh shoot/ root mass (g)	> 10^{-6} M	[33]
Castaste-rone (CS)	10^{-7}, 10^{-9} and 10^{-11} M	Cu (different concentrations)	*Brasicca juncea* 7 d old	Shoot/root (cm) length and fresh mass (mg)	≥ 10^{-7} M	[34]
24-EBL	10^{-9} - 10^{-6} M	BZR (different concentrations)	*Wolffia arrhizal* 7 d after treatment	fresh plant mass (mg)	≥ 10^{-6} M , ≤ 10^{-6} M + BZR	[35]
BL	10^{-7} M	IAA (different concentrations)	*Soybean* 4 d old	Elongation growth (mm)	> 10^{-7} M	[36]
BRs	10^{-5} M	different concentrations of Cd, glutathione, glycinebetaine, salicilic acid (GSH, GB, SA)	*rice* (japonica type) 12 d after 2nd leaf stage	Shoot/root (cm) length and fresh/ dry mass (mg)	≥ 10^{-5} M	[37]
24-EBL	10^{-6}, 10^{-8} and 10^{-10} M	Ni (different concentrations)	*Vigna radiata* (nodulated) 45 d old	Shoot/root fresh/ dry mass (g)	≥ 10^{-6} M	[38]

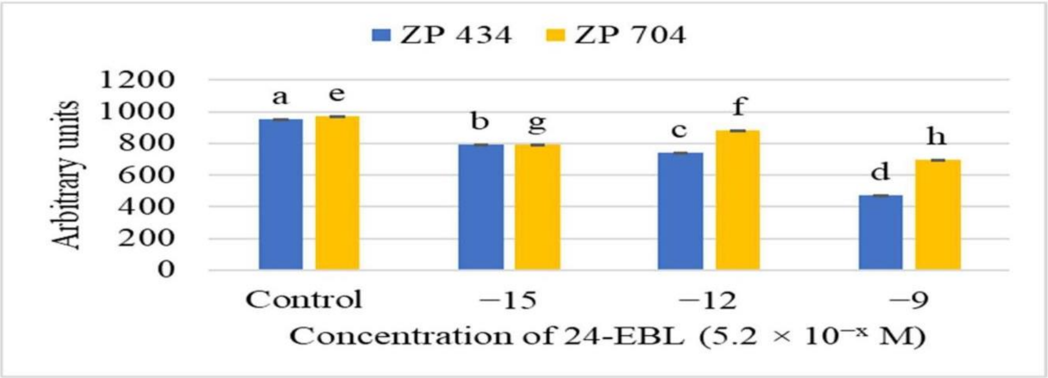

Figure S1. Effect of different concentrations of 24-EBL on total redox status of whole 7-day-old seedlings of ZP 704 and ZP 434 maize hybrids, determined by the EPR spectroscopy. The results are given in arbitrary units of the EPR signal, double integral values per sample mass. The axis values are displayed in ten thousands. Values indicated by the same letter were not statistically different ($p < 0.05$). According Božilović et al. [21].

4. **Some chemical changes during seed germination and early development and growth of a seedlings of different plant species under the influence of 24-EBL**

Table S3. Effect of different concentrations of various BRs with or without additional treatments on a photosynthetic pigments of the plant species/genotypes/ecotypes.

Type of BRs	Concentration of BRs	Other Treatments	Plant species or/ and genotypes/ ecotype; age of seedlings/ plants	Measured parameters (with unit of measure)	higher (>, ≥)/ lower (<, ≤) values then control or with non-significant difference (n-s.d.)	References
24-EBL	5.2 X 10^{-9}, 5.2 X 10^{-9} and 5.2 X 10^{-12} M	-	*maize* (ZP 434 and ZP 704)	Chla, Chlb, Chl tot, Carr (x+c) tot (mg kg^{-1} FW)	<	[22]
24-EBL	5.2 X 10^{-9}, 5.2 X 10^{-9} and 5.2 X 10^{-12} M	-	*maize* (ZP 434 and ZP 704)	Chla/b	n-s.d. (ZP 434) < (ZP 704)	[22]
24-EBL	10^{-5}, 10^{-6} and 10^{-7} M	Cold (~20/5^0C, day/night)	*maize* (line LM-17) 7, 14, 21 d after 1st leaf stage seedlings	Chl tot (mg kg^{-1} FW)	≥	[29]
24-EBL	10^{-7} M	Chlorpyrifos (0,06% dilution)	*rice* (indica type) 12 d old	Chla, Chlb, Chl tot (mg kg^{-1} FW)	<	[30]
28-HBL	10^{-9} M	Heat (40^0C)/ cold (4^0C)/ salt (180 mM)	*Brasicca juncea* 10 d old	Chla, Chlb, Chl tot, Carr (x+c) tot (mg kg^{-1} FW)	> (tretment with 28-HBL); < (tretment without 28-HBL)	[31]
24-EBL	10^{-6} - 10^{-9} M	DHECD (BRs analog; various concentrations) +heat (47^0C)	*rice* (indica type) 35+7+7 d	Chla, Chlb, Chl tot, Carr (x+c) tot (mg kg^{-1} FW)	≥/ ≤ (dependent from duration of stress)	[33]
BRs (BL, 24-EBL, 28-HBL etc.)	10^{-9} - 10^{-6} M	BZR (different concentrations)	*Wolffia arrhiza* 7 d after treatment	Chla, Chlb, different Carr (mg kg^{-1} FW)	≥ (24-EBL), ≤ (24-EBL+ BZR) < (BZR)	[35]
BRs	10^{-5} M	different concentrations of Cd, glutathione, glycinebetaine, salicilic acid (GSH, GB, SA)	*rice* (japonica type) 12 d after 2nd leaf stage	Chla, Chlb (mg kg^{-1} FW) Chl tot (SPAD value)	< (BRs+Cd) > (GSH+Cd) ≤ (GB+Cd) > (SA+Cd)	[37]
24-EBL	10^{-6}, 10^{-8} and 10^{-10} M	Ni (different concentrations)	*Vigna radiata* (nodulated) 45 d old	Chl tot (SPAD value)	≥ (24-RBL+Ni)	[38]

Table S4. Effect of different concentrations of a BRs with or without additional treatments on content of the macroelements (P, K), oligoelements (Na, Mg), microelements (Mn, Zn, Cu, Fe, Mo), and a heavy metals: (Cr, Ni, Cd) in a seedlings of various plant species/genotypes/ecotypes.

Type of BRs	Concentration of BRs	Other Treatments	Plant species or/ and genotypes/ ecotype; age of seedlings/ plants	Measured parameters (with unit of measure)	higher (>, ≥)/ lower (<, ≤) values then control or with non-significant difference (n-s.d.)	References
24-EBL	5.2 X 10^{-9}, 5.2 X 10^{-12} and 5.2 X 10^{-15} M	-	*maize* (ZP434) 7 d old	content of K (mg kg^{-1} DW) in parts (P, R, RoS)	>5.2 X 10^{-15} M (P), >5.20 X 10^{-9} M (R), <5.20 X 10^{-15} M (RoS)	[45]
24-EBL	5.2 X 10^{-9}, 5.2 X 10^{-12} and 5.2 X 10^{-15} M	-	*maize* (ZP434) 7 d old	content of K (mg kg^{-1} DW) in parts (P, R, RoS)	n-s.d. 5.2 X 10^{-9} M (P), >5.20 X 10^{-9} M (R), < 5.20 X 10^{-9} M (RoS)	[44]
24-EBL	5.2 X 10^{-9}, 5.2 X 10^{-12} and 5.2 X 10^{-15} M	-	*maize* (ZP704) 7 d old	content of K (mg kg^{-1} DW) in parts (P)	n-s.d. 5.2 X 10^{-9} M (P), >5.2 X 10^{-9} M (P)	[44,45]
24-EBL	5.2 X 10^{-9}, 5.2 X 10^{-12} and 5.2 X 10^{-15} M	-	*maize* (ZP704) 7 d old	content of K (mg kg^{-1} DW) in parts (R, RoS)	>5.20 X 10^{-9} M (R), >5.20 X 10^{-9} M (RoS)	[44,45]
24-EBL	5.2 X 10^{-9}, 5.2 X 10^{-12} and 5.2 X 10^{-15} M	-	*maize* (ZP434) 7 d old	content of Ptot (mg kg^{-1} DW) in parts (P, R, RoS)	>5.2 X 10^{-9} M (P), < 5.20 X 10^{-9} M (R), <5.20 X 10^{-12} M (RoS)	[45]
24-EBL	5.2 X 10^{-9}, 5.2 X 10^{-12} and 5.2 X 10^{-15} M	-	*maize* (ZP434) 7 d old	content of Ptot (mg kg^{-1} DW) in parts (P, R, RoS)	< 5.2 X 10^{-9} M (P), < 5.20 X 10^{-9} M (R), < 5.20 X 10^{-9} M (RoS)	[44]
24-EBL	5.2 X 10^{-9}, 5.2 X 10^{-12} and 5.2 X 10^{-15} M	-	*maize* (ZP704) 7 d old	content of Ptot (mg kg^{-1} DW) in parts (P, R, RoS)	n-s.d. 5.2 X 10^{-9} M (P), >5.20 X 10^{-9} M (R), <5.20 X 10^{-12} M (RoS)	[45]
24-EBL	5.2 X 10^{-9}, 5.2 X 10^{-12} and 5.20 X 10^{-15} M	-	*maize* (ZP704) 7 d old	content of Ptot (mg kg^{-1} DW) in parts (P, R, RoS)	> 5.2 X 10^{-9} M (P), >5.20 X 10^{-9} M (R), >5.20 X 10^{-9} M (RoS)	[44]
24-EBL	5.2 X 10^{-9}, 5.2 X 10^{-12} and 5.2 X 10^{-15} M	-	*maize* (ZP434, ZP704) 7 d old	content of Na (mg kg^{-1} DW) in parts (P, R)	> 5.2 X 10^{-9} M (P), > 5.2 X 10^{-12} M (R)	[22]
24-EBL	5.2 X 10^{-9}, 5.2 X 10^{-12} and 5.2 X 10^{-15} M	-	*maize* (ZP434, ZP704) 7 d old	content of Na (mg kg^{-1} DW) in parts (RoS)	> 5.2 X 10^{-9} M (RoS) < 5.2 X 10^{-9} M (RoS)	[22]
24-EBL	5.2 X 10^{-9}, 5.2 X 10^{-12} and 5.2 X 10^{-15} M	-	*maize* (ZP434) 7 d old	content of Mg (mg kg^{-1} DW) in parts (P, R, RoS)	≤ 5.2 X 10^{-9} M (P), ≥ 5.2 X 10^{-12} M (R), ≥ 5.2 X 10^{-12} M (RoS)	[22]
24-EBL	5.2 X 10^{-9}, 5.2 X 10^{-12} and 5.2 X 10^{-15} M	-	*maize* (ZP704) 7 d old	content of Mg (mg kg^{-1} DW) in parts (P, R, RoS)	> 5.2 X 10^{-9} M (P), > 5.2 X 10^{-9} M (R), > 5.2 X 10^{-9} M (RoS)	[22]
24-EBL	5.2 X 10^{-9}, 5.2 X 10^{-12} and 5.2 X 10^{-15} M	-	*maize* (ZP434) 7 d old	content of Mn (mg kg^{-1} DW) in parts (P, R, RoS)	< 5.2 X 10^{-12} M (P), < 5.2 X 10^{-12} M (R), < 5.2 X 10^{-12} M (RoS)	[22]

24-EBL	5.2 X 10^{-9}, 5.2 X 10^{-12} and 5.2 X 10^{-15} M	-	*maize* (ZP704) 7 d old	content of Mn (mg kg^{-1} DW) in parts (P, R, RoS)	< 5.2 X 10^{-12} M (P), >5.2 X 10^{-12} M (R), >5.2 X 10^{-15} M (RoS)	[22]
24-EBL	5.2 X 10^{-9}, 5.2 X 10^{-12} and 5.2 X 10^{-15} M	-	*maize* (ZP434) 7 d old	content of Fe (mg kg^{-1} DW) in parts (P: shoot; R: root; RoS: rest of seed)	n-s.d. 5.2 X 10^{-9} M (P), >5.2 X 10^{-15} M (R), <5.2 X 10^{-15} M (RoS)	[44,45]
24-EBL	5.2 X 10^{-9}, 5.2 X 10^{-12} and 5.2 X 10^{-15} M	-	*maize* (ZP704) 7 d old	content of Fe (mg kg^{-1} DW) in parts (P, R, RoS)	< 5.2 X 10^{-12} M (P), >5.2 X 10^{-12} M (R), >5.2 X 10^{-12} M (RoS)	[44,45]
24-EBL	5.2 X 10^{-9}, 5.2 X 10^{-12} and 5.2 X 10^{-15} M	-	*maize* (ZP434) 7 d old	content of Zn (mg kg^{-1} DW) in parts (P, R, RoS)	< 5.2 X 10^{-9} M (P), < 5.2 X 10^{-12} M (R), < 5.2 X 10^{-15} M (RoS)	[22]
24-EBL	5.2 X 10^{-9}, 5.2 X 10^{-12} and 5.2 X 10^{-15} M	-	*maize* (ZP704) 7 d old	content of Zn (mg kg^{-1} DW) in parts (P, R, RoS)	< 5.2 X 10^{-9} M (P), < 5.2 X 10^{-9} M (R), ≤ 5.2 X 10^{-9} M (RoS)	[22]
24-EBL	5.2 X 10^{-9}, 5.2 X 10^{-12} and 5.2 X 10^{-15} M	-	*maize* (ZP434) 7 d old	content of Cu (mg kg^{-1} DW) in parts (P, R, RoS)	< 5.2 X 10^{-9} M (P), < 5.2 X 10^{-12} M (R), < 5.2 X 10^{-15} M (RoS)	[22]
24-EBL	5.2 X 10^{-9}, 5.2 X 10^{-12} and 5.2 X 10^{-15} M	-	*maize* (ZP704) 7 d old	content of Cu (mg kg^{-1} DW) in parts (P, R, RoS)	< 5.2 X 10^{-9} M (P), < 5.2 X 10^{-12} M (R), < 5.2 X 10^{-12} M (RoS)	[22]
24-EBL	5.2 X 10^{-9}, 5.2 X 10^{-12} and 5.2 X 10^{-15} M	-	*maize* (ZP434) 7 d old	content of Mo (mg kg^{-1} DW) in parts (P, R, RoS)	≥ 5.2X10^{-12} M (P), ≥ 5.2X10^{-12} M (R), > 5.2X10^{-15} M (RoS)	[22]
24-EBL	5.2 X 10^{-9}, 5.2 X 10^{-12} and 5.2 X 10^{-15} M	-	*maize* (ZP704) 7 d old	content of Mo (mg kg^{-1} DW) in parts (P, R, RoS)	n-s.d. 5.2 X 10^{-9} M (P), ≥ 5.2 X 10^{-12} M (R), ≥ 5.2 X 10^{-12} M (RoS)	[22]
24-EBL	5.2 X 10^{-9}, 5.2 X 10^{-12} and 5.2 X 10^{-15} M	-	*maize* (ZP434) 7 d old	content of Cr (mg kg^{-1} DW) in parts (P, R, RoS)	< 5.2 X 10^{-9} M (P), < 5.2 X 10^{-9} M (R), < 5.2 X 10^{-9} M (RoS)	[22]
24-EBL	5.2 X 10^{-9}, 5.2 X 10^{-12} and 5.2 X 10^{-15} M	-	*maize* (ZP704) 7 d old	content of Cr (mg kg^{-1} DW) in parts (P, R, RoS)	< 5.2 X 10^{-9} M (P), < 5.2 X 10^{-9} M (R), > 5.2 X 10^{-12} M (RoS)	[22]
24-EBL	5.2 X 10^{-9}, 5.2 X 10^{-12} and 5.2 X 10^{-15} M	-	*maize* (ZP434) 7 d old	content of Ni (mg kg^{-1} DW) in parts (P, R, RoS)	n-s.d. 5.2X10^{-12} M (P), n-s.d. 5.2X10^{-12} M (R), n-s.d. 5.2X10^{-12} M (RoS)	[22]
24-EBL	5.2 X 10^{-9}, 5.2 X 10^{-12} and 5.2 X 10^{-15} M	-	*maize* (ZP704) 7 d old	content of Ni (mg kg^{-1} DW) in parts (P, R, RoS)	≤ 5.2 X 10^{-9} M (P), > 5.2 X 10^{-12} M (R), > 5.2 X 10^{-15} M (RoS)	[22]
24-EBL	5.2 X 10^{-9}, 5.2 X 10^{-12} and 5.2 X 10^{-15} M	-	*maize* (ZP434) 7 d old	content of Cd (mg kg^{-1} DW) in parts (P, R, RoS)	n-s.d. 5.2X10^{-9} M (P), n-s.d. 5.2X10^{-9} M (R), n-s.d. 5.2X10^{-9} M (RoS)	[22]
24-EBL	5.2 X 10^{-9}, 5.2 X 10^{-12} and 5.2 X 10^{-15} M	-	*maize* (ZP704) 7 d old	content of Cd (mg kg^{-1} DW) in parts (P, R, RoS)	< 5.2X10^{-9} M (P), n-s.d. 5.2X10^{-15} M (R), > 5.2X10^{-12} M (RoS)	[22]
BRs	10^{-5} M	different	*rice* (japonica type)	content of Cd (mg	(Cd treatment): >, >	[37]

		concentrations of Cd, GSH, GB, SA	12 d after 2nd leaf stage	kg^{-1} DW) in parts (P, R)	(GSH treat.): >, ><br>(BRs treat.): >, ><br>(GB treat): >, ><br>(SA treat.): >, >	
BRs	10^{-5} M	different concentrations of Cd, GSH, GB, SA	*rice* (japonica type) 12 d after 2nd leaf stage	content of Fe (mg kg^{-1} DW) in parts (P, R)	(Cd treat.): >, ><br>(GSH treat.): n-s.d., ><br>(BRs treat.): n-s.d., <<br>(GB treat.): n-s.d., n-s.d.<br>(SA treat.): >, >	[37]
BRs	10^{-5} M	different concentrations of Cd, GSH, GB, SA	*rice* (japonica type) 12 d after 2nd leaf stage	content of Mn (mg kg^{-1} DW) in parts (P, R)	(Cd treatment): <, n-s.d.<br>(GSH treat.): <, <<br>(BRs treat.): <, <<br>(GB treat): <, <<br>(SA treat.): <, n-s.d.	[37]
BRs	10^{-5} M	different concentrations of Cd, GSH, GB, SA	*rice* (japonica type) 12 d after 2nd leaf stage	content of Zn (mg kg^{-1} DW) in parts (P, R)	(Cd treatment): <, <<br>(GSH treat.): <, <<br>(BRs treat.): <, <<br>(GB treat): <, <<br>(SA treat.): <, <	[37]
BRs	10^{-5} M	different concentrations of Cd, GSH, GB, SA	*rice* (japonica type) 12 d after 2nd leaf stage	content of Cu (mg kg^{-1} DW) in parts (P, R)	(Cd treatment): ≤, <<br>(GSH treat.): ≥, <<br>(BRs treat.): ≥, <<br>(GB treat): n-s.d., <<br>(SA treat.): n-s.d., <	[37]
Castaste-rone (CS)	10^{-7}, 10^{-9} and 10^{-11} M	Cu (different concentrations)	*Brasicca juncea* 7 d old	content of Cu (mg g^{-1} DW) in parts (P)	(0 mM of Cu): n-s.d.<br>(0.25 mM of Cu): <, by 10^{-11} M of CS<br>(0.50 mM of Cu): <, by 10^{-9} M of CS<br>(0.75 mM of Cu): <, by 10^{-9} M of CS	[34]
Castaste-rone (CS)	10^{-7}, 10^{-9} and 10^{-11} M	Cu (different concentrations)	*Brasicca juncea* 7 d old	content of Cu (mg g^{-1} DW) in parts (R)	(0 mM of Cu): n-s.d.<br>(0.25 mM of Cu): <, by 10^{-7} M of CS<br>(0.50 mM of Cu): <, by 10^{-7} M of CS<br>(0.75 mM of Cu): <, by 10^{-9} M of CS	[34]
24-EBL	10^{-7}, 10^{-9} and 10^{-11} M	Zn, Mn, Co, Ni (different concentrations)	*Brasicca juncea* 7 d old	Bioconcentration factor (BCF): heavy metals content in plants (mg kg^{-1} FW)/ heavy metals concentration in solution (mg/l)	BCF of Zn: < by 10^{-7} M<br>BCF of Mn: < by 10^{-9} M<br>BCF of Co: < by 10^{-9} M<br>BCF of Ni: < by 10^{-7} M	[32]
28-HBL	10^{-7}, 10^{-9} and	Zn (different	*Brasicca juncea*	Bioconcentration	BCF of Zn: n-s.d.	[32]

	10^{-11} M	concentrations)	7 d old	factor (BCF)	by 10^{-9} M	
3028-HBL	10^{-7}, 10^{-9} and 10^{-11} M	Zn (different concentrations)	*Brasicca juncea* 7 d old	Zink metal uptake (mg g^{-1} DW)	BCF of Zn: < by 10^{-9} M	[32]

Table S5. Effect of different concentrations of various BRs with or without additional treatments on content of some sugars in a seedlings of different plant species/genotypes/ecotypes.

Type of BRs	Concentration of BRs	Other Treatments	Plant species or/ and genotypes/ ecotype; age of seedlings/ plants	Measured parameters (with unit of measure)	higher (>, ≥)/ lower (<, ≤) values then control or with non-significant difference (n-s.d.)	References
24-EBL	5.2 X 10^{-9}, 5.2 X 10^{-12} and 5.2 X 10^{-15} M	-	*maize* (ZP434, ZP704) 7 d old	Content of Tre sugar (mg kg^{-1} DW)	ZP434: > 5.2 X 10^{-15} M (P); < 5.2 X 10^{-9} M (R); > 5.2 X 10^{-15} M (RoS); ZP434: > 5.2 X 10^{-15} M (P, R, RoS)	[45]
24-EBL	5.2 X 10^{-9}, 5.2 X 10^{-12} and 5.2 X 10^{-15} M	-	*maize* (ZP434, ZP704) 7 d old	Content of Glu sugar (mg kg^{-1} DW)	ZP434: < 5.2 X 10^{-15} M (P, RoS); < 5.2 X 10^{-9} M (R); ZP704: > 5.2 X 10^{-9} M (P); > 5.2 X 10^{-12} M (R); < 5.2 X 10^{-9} M (RoS)	[45]
24-EBL	5.2 X 10^{-9}, 5.2 X 10^{-12} and 5.2 X 10^{-15} M	-	*maize* (ZP434, ZP704) 7 d old	Content of Fru sugar (mg kg^{-1} DW)	ZP434: < 5.2 X 10^{-9} M (P); < 5.2 X 10^{-15} M (R,RoS); ZP704: > 5.2 X 10^{-9} M (P, RoS); > 5.2 X 10^{-15} M (R);	[45]
24-EBL	5.2 X 10^{-9}, 5.2 X 10^{-12} and 5.2 X 10^{-15} M	-	*maize* (ZP434, ZP704) 7 d old	Content of Suc sugar (mg kg^{-1} DW)	ZP434: >5.2 X 10^{-12} M (P, RoS); > 5.2 X 10^{-15} M (R); ZP704: > 5.2 X 10^{-12} M (P, RoS); < 5.2 X 10^{-9} M (R);	[45]
24-EBL	5.2 X 10^{-9}, 5.2 X 10^{-12} and 5.2 X 10^{-15} M	-	*maize* (ZP434, ZP704) 7 d old	Content of Raf sugar (mg kg^{-1} DW)	ZP434: >5.2 X 10^{-9} M (P, R); < 5.2 X 10^{-9} M (RoS); ZP704: > 5.2 X 10^{-12} M (P); > 5.2 X 10^{-9} M (R, RoS);	[44]
24-EBL	5.2 X 10^{-9}, 5.2 X 10^{-12} and 5.2 X 10^{-15} M	-	*maize* (ZP434, ZP704) 7 d old	Content of Tre sugar (mg kg^{-1} DW)	ZP434: >5.2 X 10^{-9} M (P); < 5.2 X 10^{-9} M (R); > 5.2 X 10^{-15} M (RoS); ZP704: > 5.2 X 10^{-15} M (P, R,	[44]

					RoS);	
24-EBL	5.2 X 10^{-9},<br>5.2 X 10^{-12} and<br>5.2 X 10^{-15} M	-	*maize*<br>(ZP434, ZP704)<br>7 d old	Content of<br>Glu sugar<br>(mg kg^{-1} DW)	ZP434:<br>< 5.2X10^{-15} M (P, RoS);<br>< 5.2 X 10^{-9} M (R);<br>ZP704:<br>> 5.2 X 10^{-9} M (P, R);<br>< 5.2X10^{-15} M (RoS);	[44]
24-EBL	5.2 X 10^{-9},<br>5.2 X 10^{-12} and<br>5.2 X 10^{-15} M	-	*maize*<br>(ZP434, ZP704)<br>7 d old	Content of<br>Fru sugar<br>(mg kg^{-1} DW)	ZP434:<br>< 5.2X10^{-9} M (P);<br>< 5.2X10^{-15} M (R, RoS);<br>ZP704:<br>> 5.2 X 10^{-9} M (P, RoS);<br>> 5.2 X 10^{-12} M (R);	[44]
24-EBL	5.2 X 10^{-9},<br>.2 X 10^{-12} and<br>5.2 X 10^{-15} M	-	*maize*<br>(ZP434, ZP704)<br>7 d old	Content of<br>Suc sugar<br>(mg kg^{-1} DW)	ZP434:<br>> 5.2X10^{-12} M (P, R);<br>> 5.2X10^{-15} M (RoS);<br>ZP704:<br>> 5.2 X 10^{-12} M (P);<br>< 5.2 X 10^{-9} M (R);<br>> 5.2 X 10^{-9} M (RoS);	[44]
24-EBL	10^{-5}, 10^{-6}<br>and 10^{-7} M	Cold (~20/5^{0}C, day/night)	*maize* (line LM-17)<br>7, 14, 21 d after 1st leaf stage seedlings	Content of Glu and Suc sugars<br>(mg g^{-1} FW)	Influence on Glu and Suc content:<br>Control& cold treatment:<br>> 10^{-6} M	[29]
24-EBL	10^{-6} - 10^{-9} M	DHECD (BRs analog; various concentrations)<br>+heat (47^{0}C)	*rice* (indica type)<br>35+7+7 d	Content of total soluble sugars<br>(mg g^{-1} FW)	Stress control: <<br>24-EBL+stress: <<br>DHECD+stress: <	[33]
BRs (BL, 24-EBL, 28-HBL etc.)	10^{-9} - 10^{-6} M	BZR (different concentrations)	*Wolffia arrhiza*<br>7 d after treatment	Content of monosacharide sugars<br>(mg g^{-1} FW)	BZR treat.: < 10^{-5} M<br>BRs treat.: > 10^{-7} M<br>BZR+BRs treat.: ≤	[35]

Figure S2. Effect of different concentrations of 24-EBL on HPTLC chromatograms of maize extracts for ZP 434 (a, b) and ZP 704 (c, d) hybrids obtained in CS1 and CS2 methods, respectively. According Waisi et al. [55].

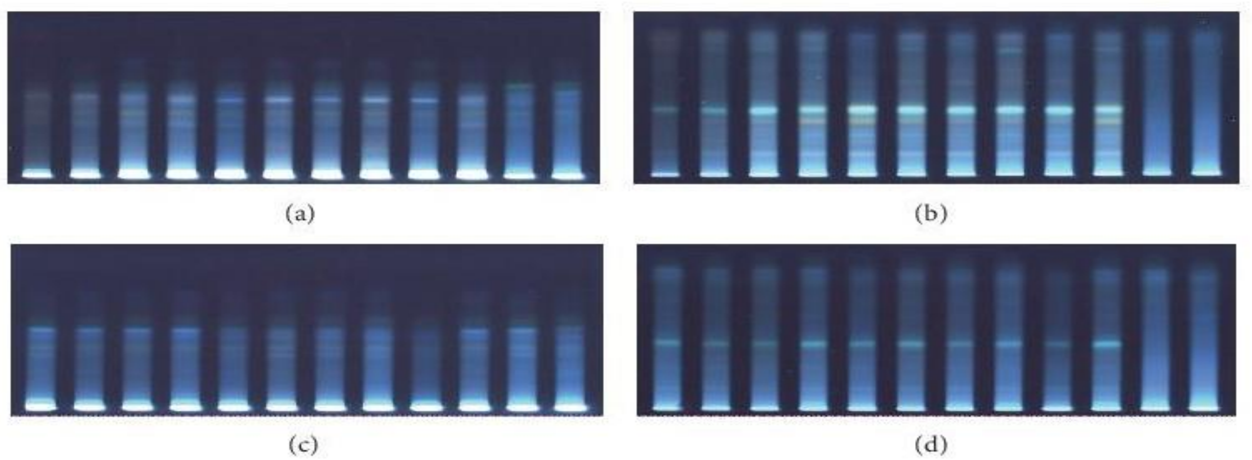

Table S6. Order of samples in chromatograms sequence on each plate. For practical reasons, the complete polyphenolic profile of the control and all treated plumule (shoot) samples, as well as control samples of radicles (root) and radicles treated with 24-EBL concentration 5.2 X 10^{-11} M is presented. According Waisi et al. [55].

number	Treatment of maize seedling part (Plumule:S; Radicle: R	label
1	Shoot treated with 5.2 X 10^{-7} M of BRs	S-7
2	Shoot treated with 5.2 X 10^{-8} M of BRs	S-8
3	Shoot treated with 5.2 X 10^{-9} M of BRs	S-9
4	Shoot treated with 5.2 X 10^{-10} M of BRs	S-10
5	Shoot treated with 5.2 X 10^{-11} M of BRs	S-11
6	Shoot treated with 5.2 X 10^{-12} M of BRs	S-12
7	Shoot treated with 5.2 X 10^{-13} M of BRs	S-13
8	Shoot treated with 5.2 X 10^{-14} M of BRs	S-14
9	Shoot treated with 5.2 X 10^{-15} M of BRs	S-15
10	Shoot-control	Sc
11	Root-control	Rc
12	Root treated with 5.2 X 10^{-11} M of BRs	R-11

Figure S3. The line profile plots of chromatograms of shoot control samples of ZP 434 (a) and ZP 704 (b) hybrids obtained in CS2 and split through RGB channels (R: red, G: green, and B: blue). Labels on score plot correspond to those in Table S6. According Waisi et al. [55].

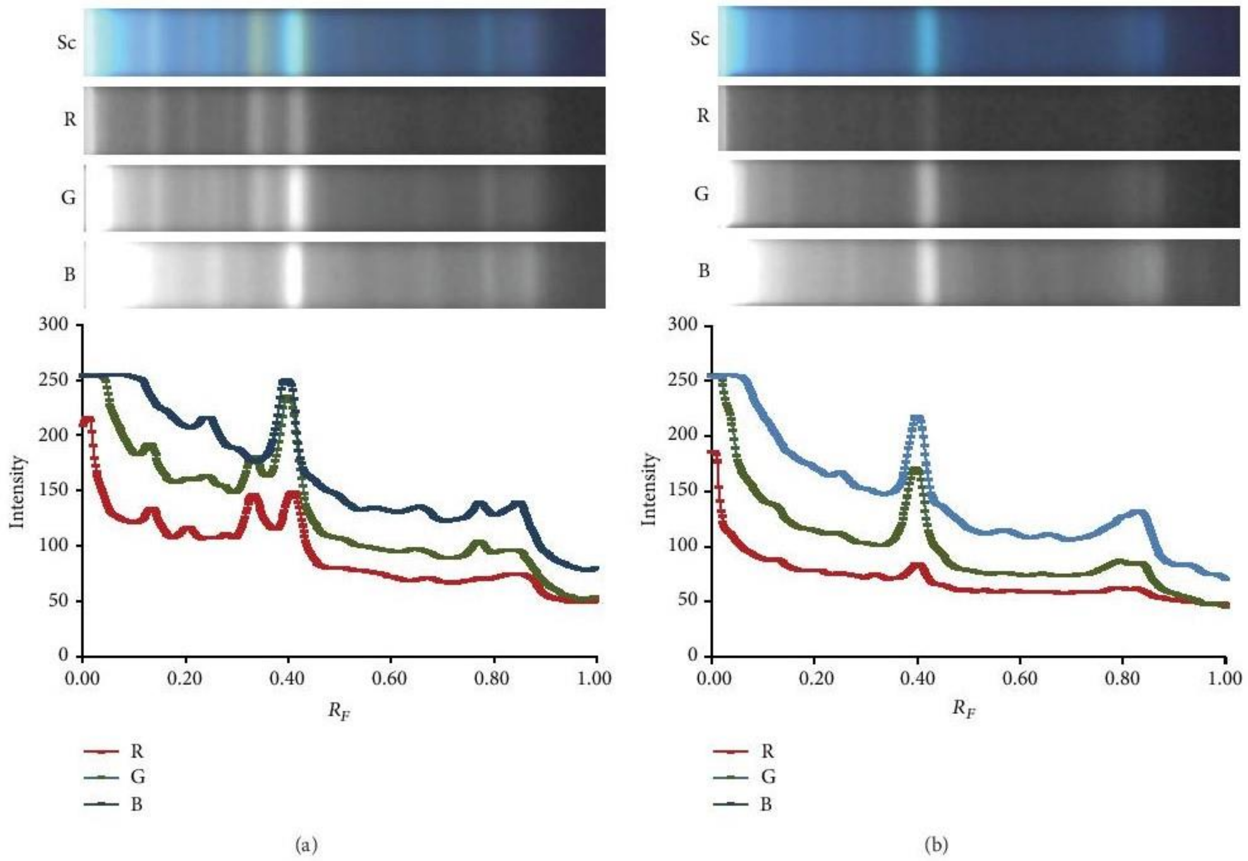

Figure S4. Phenolic profiles of shoot and root control samples for ZP 434 (a, c) and ZP 704 (b, d) hybrids obtained in CS2 and CS1, respectively, and shoot samples of ZP 434 (e) and ZP 704 (f) hybrids treated with brassinosteroid type of phytohormone in concentration of 5.2 $\times 10^{-7}$ – 5.2 $\times 10^{-15}$ M. Labels on score plot correspond to those in Table S6. According Waisi et al. [55].

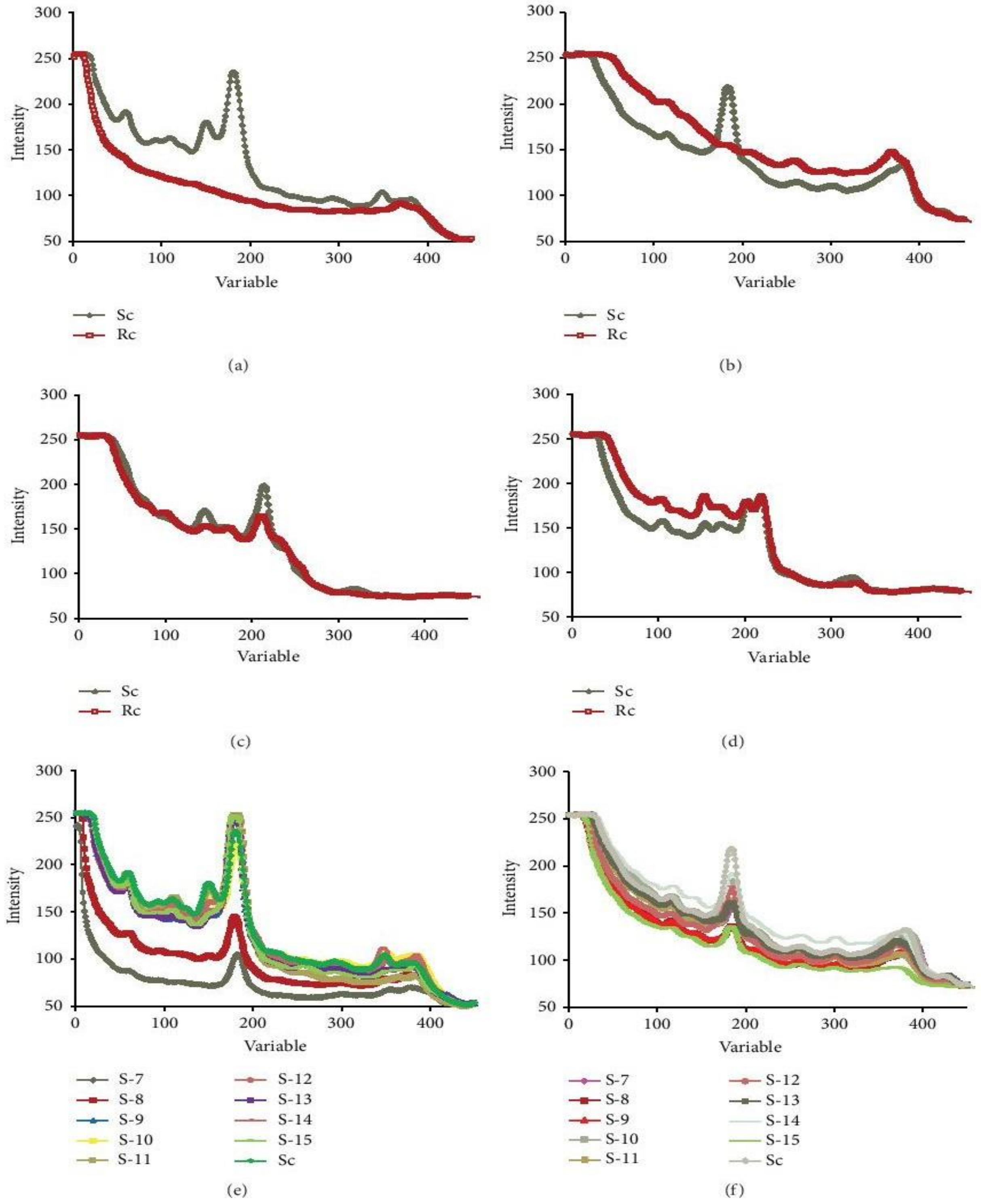

Figure S5. PCA analysis performed on data obtained in CS1 method. (a) mutual projections of factor scores, and (b, c) loadings for the PC1 and PC2, respectively, for a model corresponding to blue channel (see: Figures S2, S3, S4). Labels on score plot correspond to those in Table S6. Figures according Waisi et al. [55].

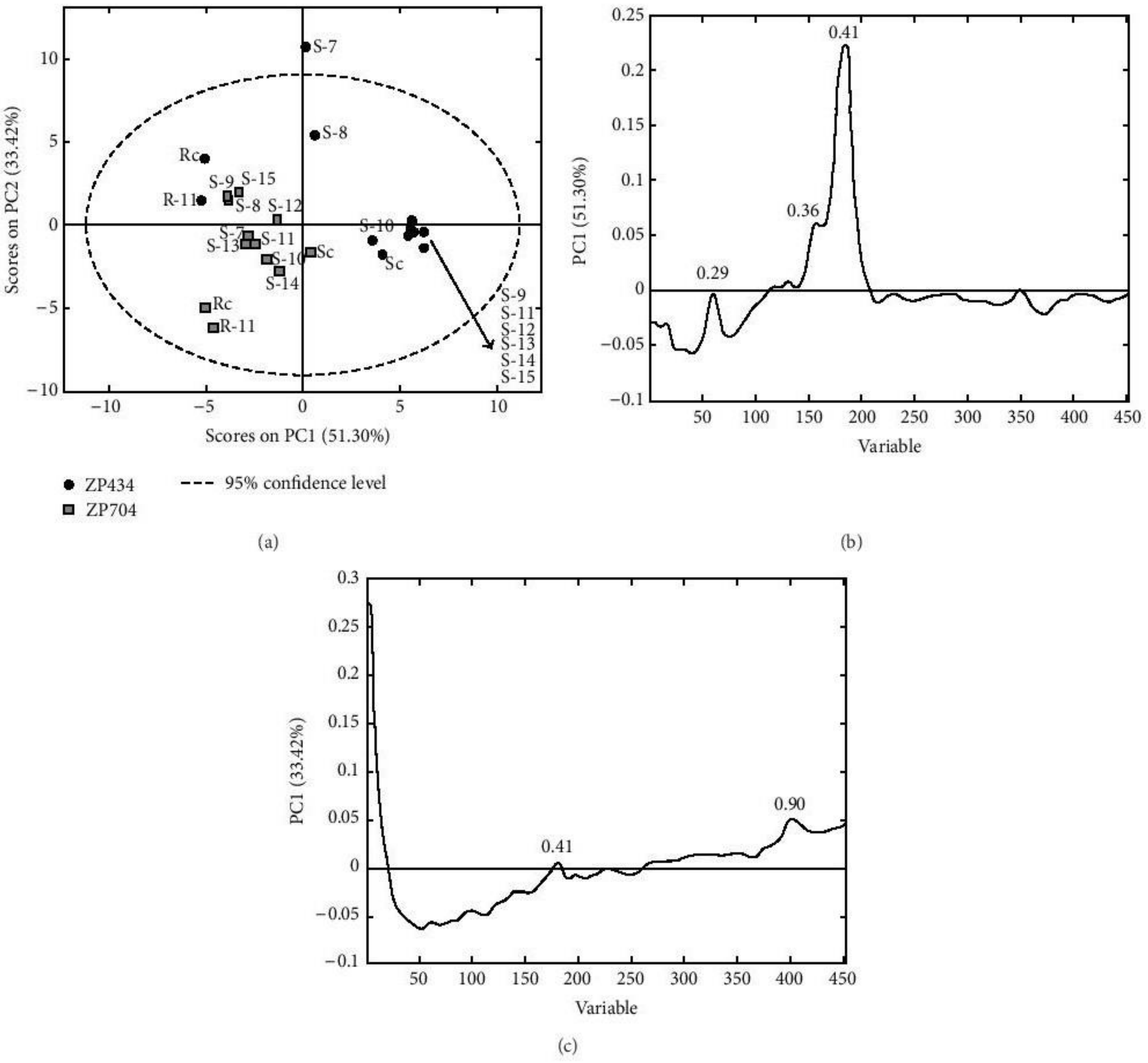

Figure S6. PCA analysis performed on data obtained in CS2 method. (a) mutual projections of factor scores, and (b, c) loadings for the PC1 and PC2, respectively, for a model corresponding to blue channel (see: Figures S2, S3, S4). Labels on score plot correspond to those in Table S6. Figures according Waisi et al. [55].

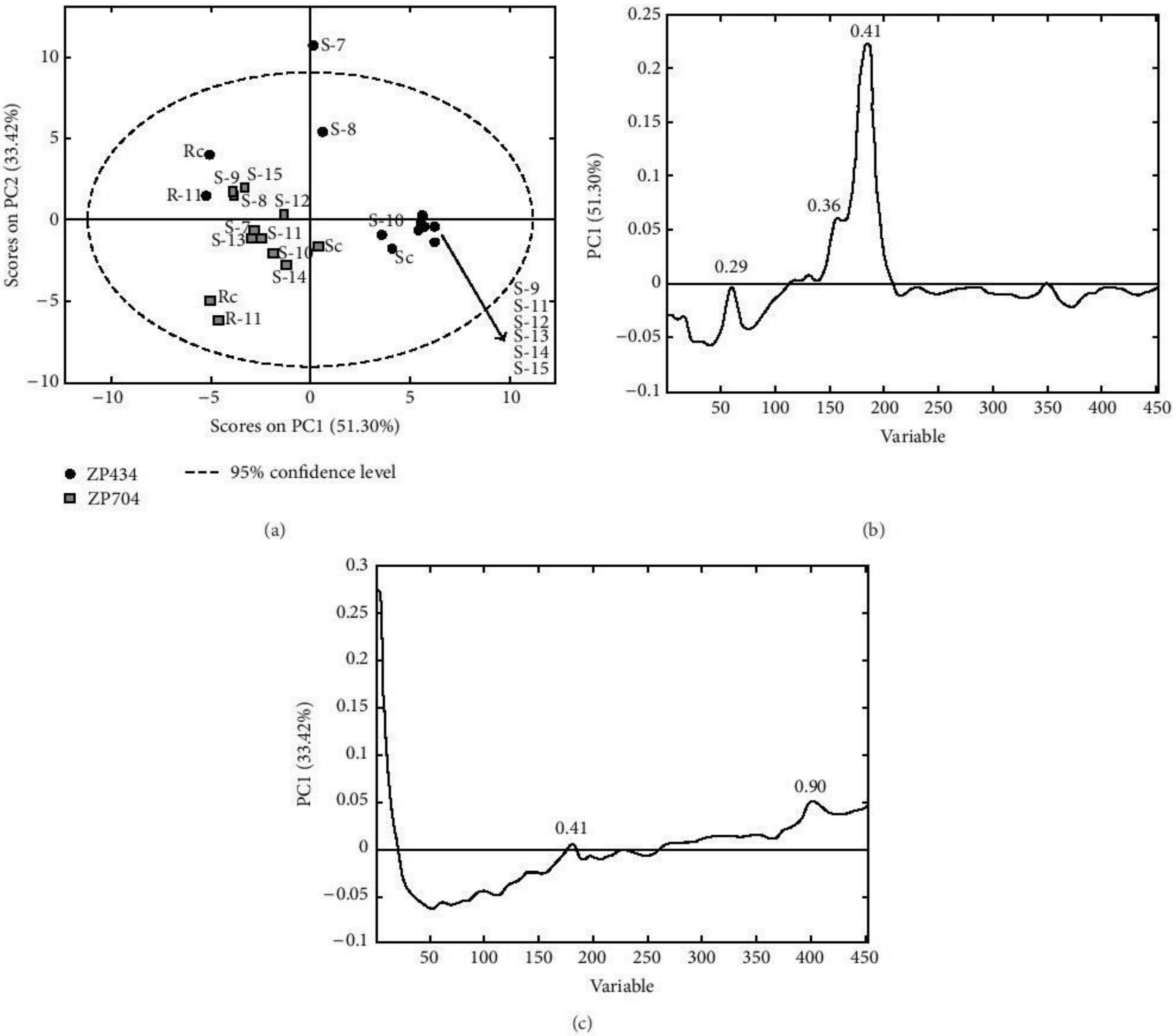

Table S7. Statistical parameters of the models of regression relationship between the content of selected sugars (Tre, Ara, Glu, Fru, Suc, Raf) and Gibbs free energy assessed in 7-day-old maize seedlings (hybrid ZP 434) and their parts. According Božilović et al. [21].

**Thermodynamic parameter**	**Seedling parts**	**Model parameters**	**Regression coefficients in model***
Entropy	Plumula (P)	Low statistical significance model	
	Radicula (R)	*RMSEC*: 0.0020<br>*RMSECV*: 0.0046<br>R^2_{Cal}: 0.7561<br>R^2_{CV}: 0.2554	Tre (+), Suc (+), Ara (+)<br>Glu (-), Fru (-)<br>Raf (0)
	Rest of seed (RoS/S)	Low statistical significance model	
	Whole seedling	Low statistical significance model	
Enthalpy	Plumula (P)	Low statistical significance model	
	Radicula (R)	*RMSEC*: 0.3711<br>*RMSECV*: 0.8215<br>R^2_{Cal}: 0.7563<br>R^2_{CV}: 0.2823	Fru (+), Glu (+)<br>Ara (-), Suc (-), Tre (-)<br>Raf (0)
	Rest of seed (RoS/S)	Low statistical significance model	
	Whole seedling	Low statistical significance model	
Gibbs Free Energy	Plumula (P)	*RMSEC*: 0.0152<br>*RMSECV*: 0.0539<br>R^2_{Cal}: 0.9714<br>R^2_{CV}: 0.8479	Raf (+), Tre (+), Fru (+), Glu (+)<br>Ara (-), Suc (-)
	Radicula (R)	*RMSEC*: 0.0357<br>*RMSECV*: 0.0454<br>R^2_{Cal}: 0.8517<br>R^2_{CV}: 0.7779	Glu (+), Fru (+), Tre (+), Suc (+), Raf (+), Ara (+)
	Rest of seed (RoS/S)	*RMSEC*: 0.1866<br>*RMSECV*: 0.2947<br>R^2_{Cal}: 0.6471<br>R^2_{CV}: 0.3319	Suc (+), Raf (+)<br>Ara (-), Glu (-), Fru (-), Tre (-)
	Whole seedling	*RMSEC*: 0.5123<br>*RMSECV*: 0.5931<br>R^2_{Cal}: 0.7188<br>R^2_{CV}: 0.6328	Glu (+), Ara (+), Tre (+)<br>Raf (-), Suc (-), Fru (-)

* + (positive influence on the dependent variable, in descending order);
– (negative influence on the dependent variable, in descending order)

Table S8. Model parameters of regression relationship between the content of selected sugars (Tre, Ara, Glu, Fru, Suc, Raf) and differential thermodynamic parameters (entropy, enthalpy, and Gibbs free energy) assessed in 7-day-old maize seedlings (hybrid ZP 704) and their parts. According Božilović et al. [21].

**Thermodynamic parameter**	**Seedling parts**	**Model parameters**	**Regression coefficients in model***
Entropy	Plumula (P)	*RMSEC*: 0.0072<br>*RMSECV*: 0.0098<br>R^2_{Cal}: 0.7339<br>R^2_{CV}: 0.5425	Raf (+)<br>Glu (-), Suc (-), Ara (-), Tre (-)<br>Fru (0)
	Radicula (R)	*RMSEC*: 0.0087<br>*RMSECV*: 0.0097<br>R^2_{Cal}: 0.6509<br>R^2_{CV}: 0.6180	Raf (+), Fru (+), Glu (+), Suc (+)<br>Ara (-), Tre (-)
	Rest of seed (RoS/S)	*RMSEC*: 0.0077<br>*RMSECV*: 0.0102<br>R^2_{Cal}: 0.8687<br>R^2_{CV}: 0.8117	Glu (+), Ara (+), Suc (+), Raf (+), Fru (+)<br>Tre (-)
	Whole seedling	Low statistical significance model	
Enthalpy	Plumula (P)	*RMSEC*: 1.3083<br>*RMSECV*: 1.7839<br>R^2_{Cal}: 0.7340<br>R^2_{CV}: 0.5433	Tre (+), Ara (+), Suc (+), Glu (+), Raf (-)<br>Fru (0)
	Radicula (R)	*RMSEC*: 1.5849<br>*RMSECV*: 1.7754<br>R^2_{Cal}: 0.6508<br>R^2_{CV}: 0.5632	Tre (+), Ara (+)<br>Suc (-), Glu (-), Fru (-), Raf (-)
	Rest of seed (RoS/S)	*RMSEC*: 1.4122<br>*RMSECV*: 2.0551<br>R^2_{Cal}: 0.8687<br>R^2_{CV}: 0.7782	Tre (+)<br>Fru (-), Suc (-), Raf (-), Glu (-), Ara (-)
	Whole seedling	Low statistical significance model	
Gibbs Free Energy	Plumula (P)	*RMSEC*: 0.0709<br>*RMSECV*: 0.1692<br>R^2_{Cal}: 0.3456<br>R^2_{CV}: 0.4042	Tre (+), Raf (+), Fru (+), Ara (+), Glu (+)<br>Suc (-)
	Radicula (R)	Low statistical significance model	
	Rest of seed (RoS/S)	*RMSEC*: 0.0904<br>*RMSECV*: 0.2579<br>R^2_{Cal}: 0.8282<br>R^2_{CV}: 0.4600	Suc (+), Ara (+), Fru (+), Tre (+)<br>Glu (-), Raf (-)
	Whole seedling	*RMSEC*: 0.5291<br>*RMSECV*: 0.6804<br>R^2_{Cal}: 0.8075<br>R^2_{CV}: 0.7112	Glu (+), Ara (+),<br>Tre (-), Raf (-), Suc (-), Fru (-)

* + (positive influence on the dependent variable, in descending order);
– (negative influence on the dependent variable, in descending order)

Figure S7. Changes of values of ΔH* as a function of changes of values of ΔS* (according equation: $\Delta G^* = \Delta H^* - T_i \Delta S^*$) [14], for processes of dehydratation of seedlings of ZP434 maize hybrids. Similar to Waisi [40].

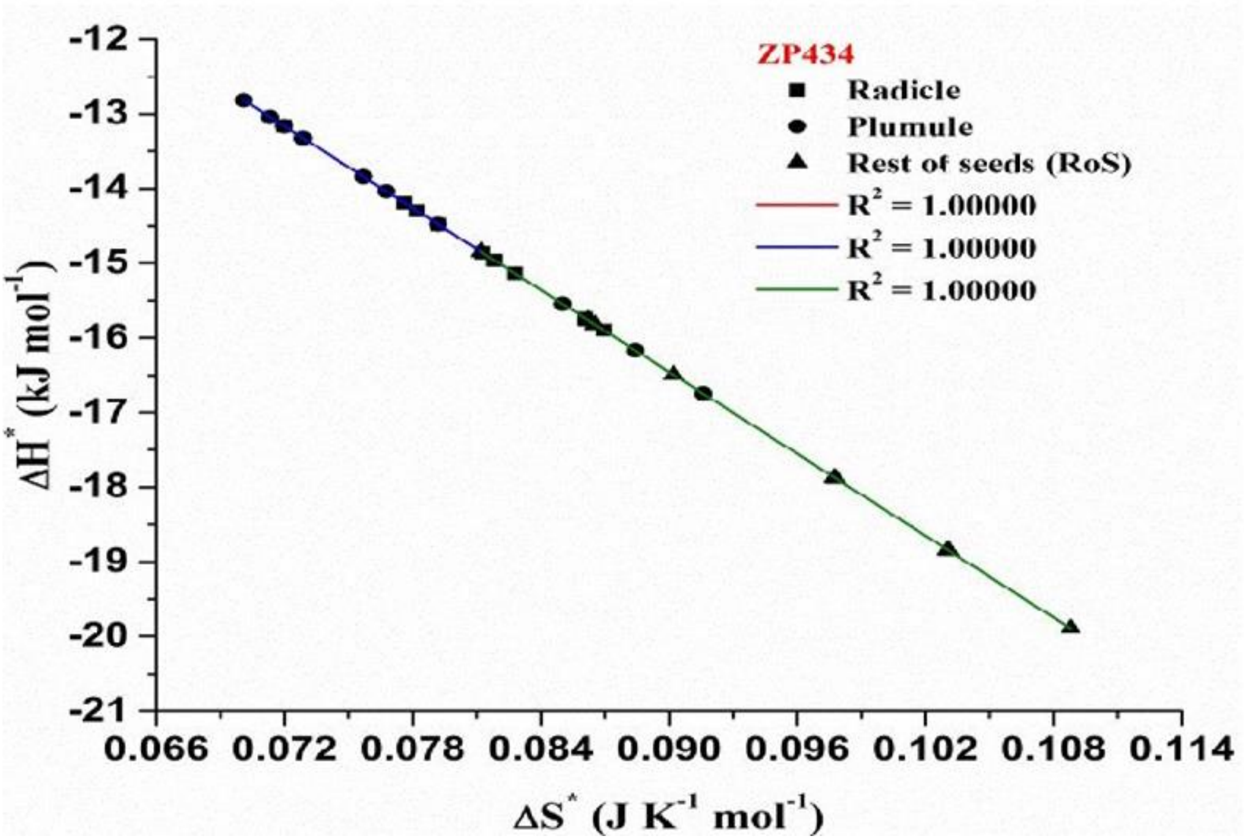

Figure S8. Changes of values of ΔH* as a function of changes of values of ΔS* (according equation: ΔG*= ΔH* - T_iΔS*) [14], for processes of dehydratation of seedlings of ZP704 maize hybrids. Similar to Waisi [40].

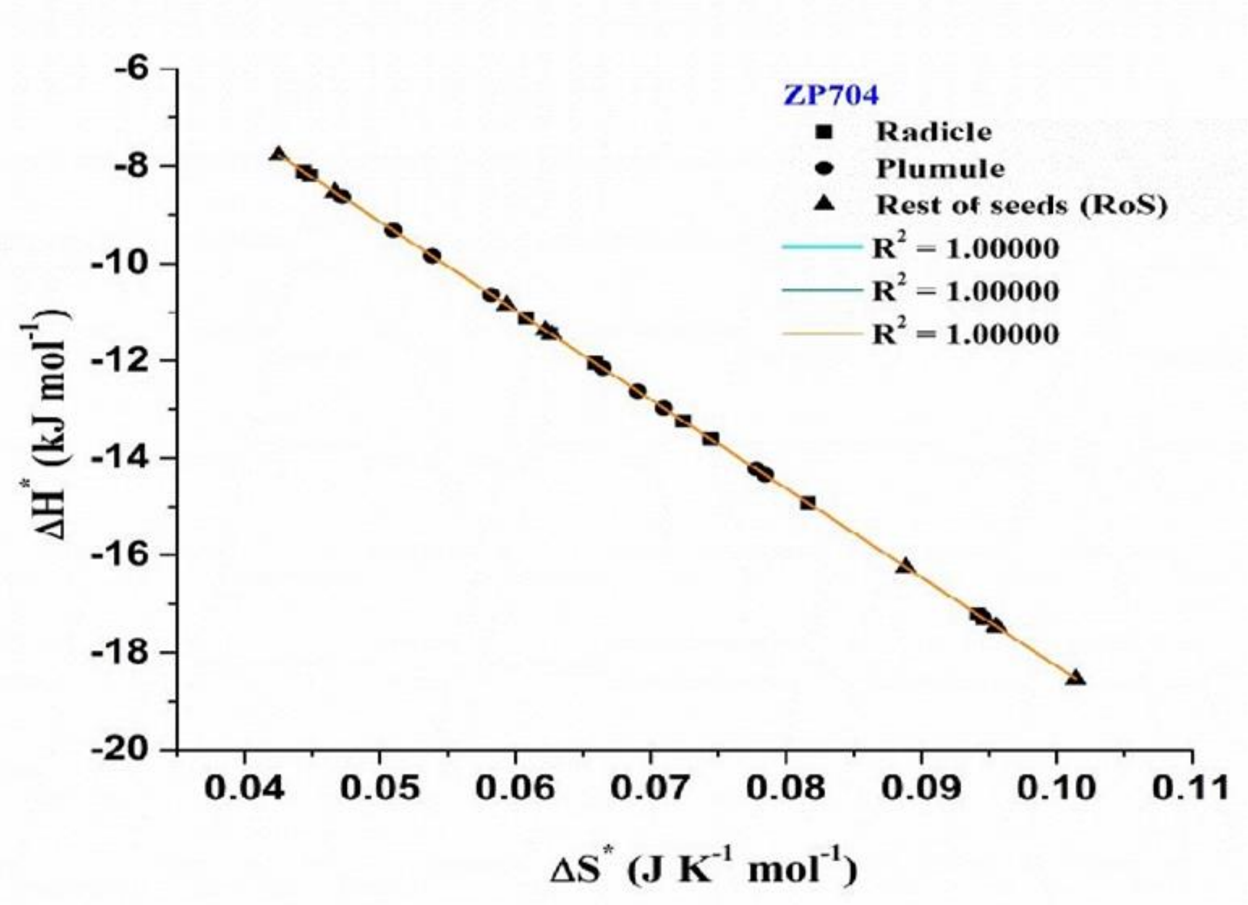

Supplement: Supplementary file 1 [file ijms-26-02559-s001.zip › ijms-3431492-supplementary.pdf]
